# Double-Layered Lateral Meniscus in an 8-Year-Old Child: Report of a Rare Case

**DOI:** 10.1155/2016/5263248

**Published:** 2016-10-19

**Authors:** Susumu Araki, Mitsuhiko Kubo, Kosuke Kumagai, Shinji Imai

**Affiliations:** ^1^Department of Orthopaedic Surgery, Shiga University of Medical Science, Otsu, Shiga 520-2192, Japan; ^2^Public Interest Incorporated Foundation, Toyosato Hospital, No. 12, 8 Moku, Toyosato-cho, Inukami, Shiga 529-1158, Japan

## Abstract

Reports of congenital abnormalities of the lateral meniscus include discoid meniscus, accessory meniscus, double-layered meniscus, and ring-shaped meniscus. Particularly, only a few cases of double-layered meniscus have been reported. We report a case of double-layered lateral meniscus, in which an additional semicircular meniscus was observed under the normal lateral meniscus. The accessory hemimeniscus was resected by means of arthroscopic surgery. This case demonstrates an interesting and extremely rare anatomical abnormality of the lateral meniscus.

## 1. Introduction

The overall incidence of meniscal anomalies is rather small. These anomalies are more frequent in East Asian populations and tend to be more prevalent in the lateral meniscus. Discoid meniscus is the most common aberration, but other meniscus malformations are rare [1]. Among them, cases of accessory lateral meniscus in the form of a double-layered meniscus are extremely uncommon. To our knowledge, only a few such cases have been described to date [2–10]. Furthermore, our report involves the second youngest case patient thus far [10]. We report on a rare case that we diagnosed as double-layered lateral meniscus and operated on the patient by performing partial resection with arthroscopy. A physical examination of the left knee revealed pain and catching at the lateral aspect on deep flexion.

## 2. Case Report

### 2.1. History of Present Illness

**Figure 1:**
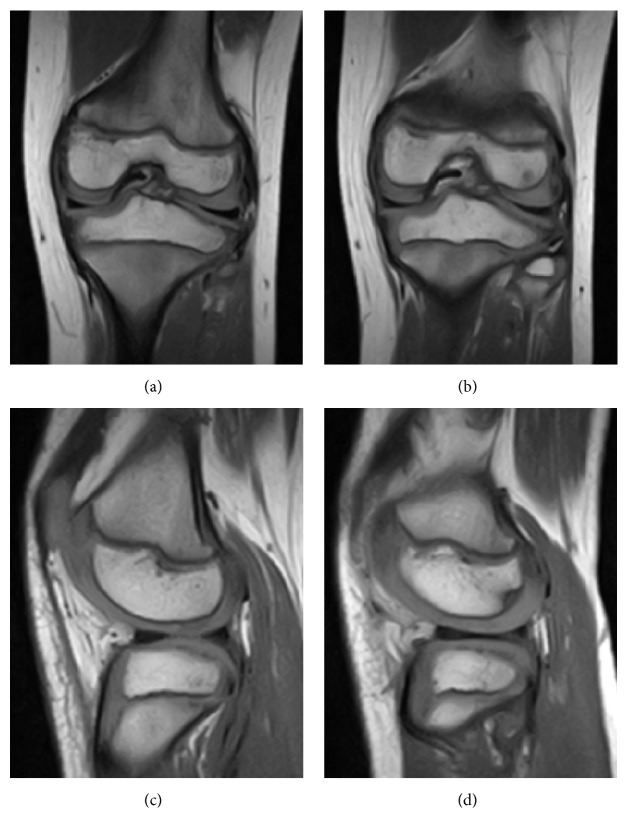
T1-weighted magnetic resonance images. Coronal (a, b) and sagittal (c, d) sections showing normal findings of the lateral meniscus.

An 8-year-old boy presented with pain in the left knee during baseball practice. He had no history of knee injury. At presentation at our institution (1 week after the onset of pain), his left knee pain and hydrarthrosis had become too severe to continue playing baseball well. On physical examination, full range of motion, tenderness at the lateral joint line, pain and catching on deep flexion, and a positive McMurray test on the lateral side were observed. His gait was normal and there was no sign of giving way of the affected knee. The valgus and varus stress tests, anterior drawer test, pivot-shift test, and posterior drawer test had negative results, indicating an absence of ligamentous abnormalities. The neurovascular examination showed normal findings. The radiographs and magnetic resonance images (MRIs) were normal (Figure 1).

### 2.2. Past Medical History

There was no history of injury and any other illness.

### 2.3. Family History

There was no relevant family history.

### 2.4. Athletic Experience

The patient had been playing baseball (position: catcher) for 3 years.

### 2.5. Surgical Operation and Postoperative Course

**Figure 2:**
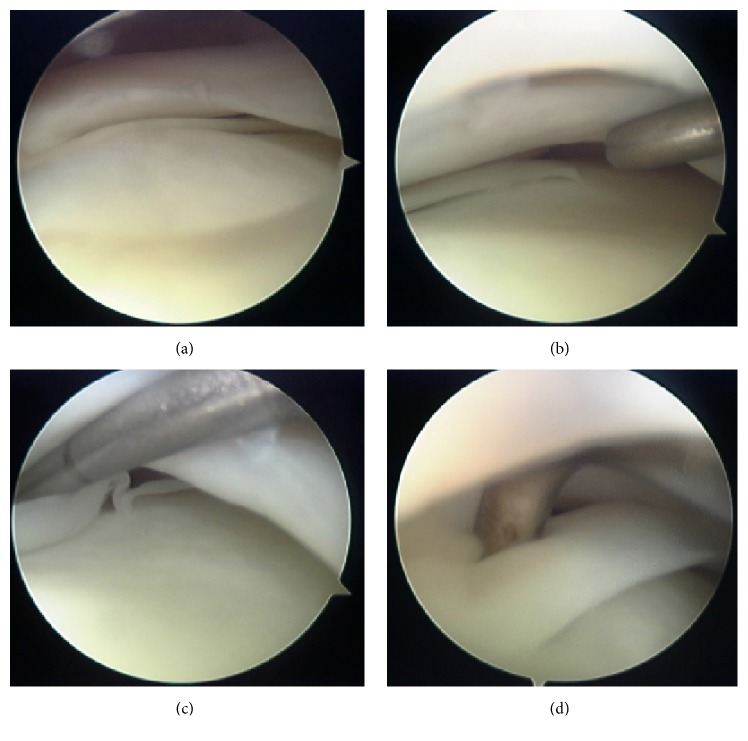
Arthroscopic findings. (a) Lateral meniscus of the left knee. (b) A second semicircular meniscus is seen under the original lateral meniscus. It could be pulled forward easily on probing (c, d).

We performed follow-up examination on the patient for 2 weeks; however, his symptoms did not improve (especially the knee pain and catching on deep flexion). Therefore, he underwent arthroscopy of the left knee. On examination of the lateral compartment, a second semicircular meniscus was seen under the original lateral meniscus, and it could be pulled forward easily on probing (Figure 2). There were no other joint abnormalities. The second semicircular meniscus of the left knee was resected arthroscopically, resulting in the complete relief of symptoms. The patient has now been able to return to baseball practice without restrictions.

The patient and his family members were informed that data from the case would be submitted for publication and provided their consent.

### 2.6. Histological Findings

**Figure 3:**
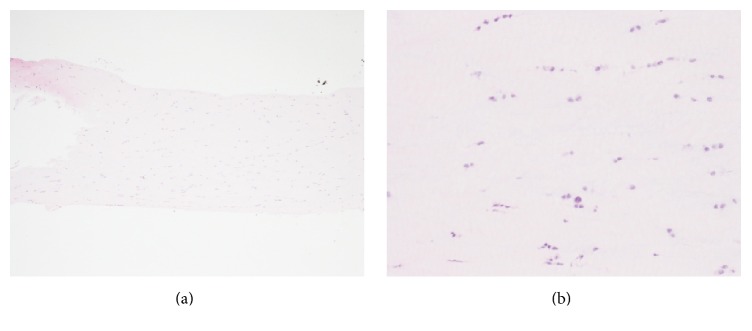
Histological findings of the second semicircular meniscus. No disorder was seen in the arrangement of collagen fibers, which were dense and parallel. Hematoxylin and eosin staining, ×40 (a) and ×100 (b).

In the lower layer of the meniscus, no disorder was seen in the arrangement of collagen fibers, which were dense and parallel (Figure 3).

## 3. Discussion

After the operation, we rechecked the previous MRIs; however, we could not find any lesion from those images. However, before the operation, the patient had been experiencing sharp pain and catching of the posterolateral side of his left knee on deep flexion. This finding made us suspect the presence of a meniscus lesion (e.g., lateral meniscus injury or a hypermobile meniscus), a synovium mass lesion, or a cartilage lesion. Furthermore, the possibility of an inflammatory lesion from his knee hydrarthrosis was considered. However, in this case, we judged it to be absent because the sharp pain and catching of the left knee occur only during motion. The patient frequently played baseball as a catcher. This position often requires a full range of motion of the knee. Therefore, we considered that this fact was closely related to the symptoms.

In this case, as mentioned above, a second semicircular meniscus was seen under the original lateral meniscus. Furthermore, the middle part of the abnormal meniscus was attached to the normal meniscus and the posterior horn was attached to the tibial articular cartilage surface. Concerning the movement of a normal lateral meniscus, it is believed that the lateral femoral condyle rolls back in the knee flexure, and at the same time the lateral meniscus recedes following the femoral condyle. However, in this case, the patient felt clicking and pain in the left knee because his abnormal meniscus was attached to the tibial plateau and it was left against the lateral femoral condyle.

A discoid lateral meniscus is the most common abnormality of the lateral meniscus. However, cases of accessory lateral meniscus in the form of a double-layered meniscus are extremely uncommon (reported with a prevalence of 0.06% to 0.09% [2, 3]) and, when present, are believed to potentially contribute to the symptoms of patients [2]. To our knowledge, only nine such cases have been described to date [2–10]. All patients were young and had malformation of the lateral meniscus. Furthermore, in all nine cases, an accessory meniscus was overlying the normal lateral meniscus. This case demonstrates an interesting and extremely rare anatomical abnormality of the lateral meniscus. This is the first reported case of an accessory meniscus under a layered original meniscus.

As with other meniscus malformations, a double-layered meniscus is believed to develop in the early fetal periods [11]. At the age of 8 years, the present patient is the second youngest case patient in the literature [10]. The age at presentation of the patient coincides with the start of competition activities in school club teams in Japan and thus correlates with an increase in physical activity compared with children in lower grades. Furthermore, it was considered that the nature of the movements of the patient as a baseball catcher induced his symptoms. The presence of an abnormally shaped meniscus may lead to significantly altered biomechanics of the lateral compartment. The clinical significance of such altered biomechanics is not always clear; however, they have often been associated with pain and clicking. In addition, it is clinically important to differentiate such symptoms from any other pain and/or clicking in the knee in children.

## 4. Conclusions

We reported a case of double-layered lateral meniscus in the second youngest patient thus far. Furthermore, this is the first reported case of an accessory meniscus located under the normal lateral meniscus. Arthroscopic partial meniscectomy resulted in patient satisfaction and a return to normal daily activities, which included baseball practice. In the case of suspicion of a meniscal lesion or its symptoms on physical examination, meniscal malformation/injury should be considered even if the MRI does not show any injury/morphological aberration.
